# Supplementary figures and images for: Sodium–calcium exchanger 1 is the key molecule for urinary potassium excretion against acute hyperkalemia
Source: PLoS One. 2020 Jun 30;15(6):e0235360. doi: 10.1371/journal.pone.0235360 (PMC7326190; doi:10.1371/journal.pone.0235360)

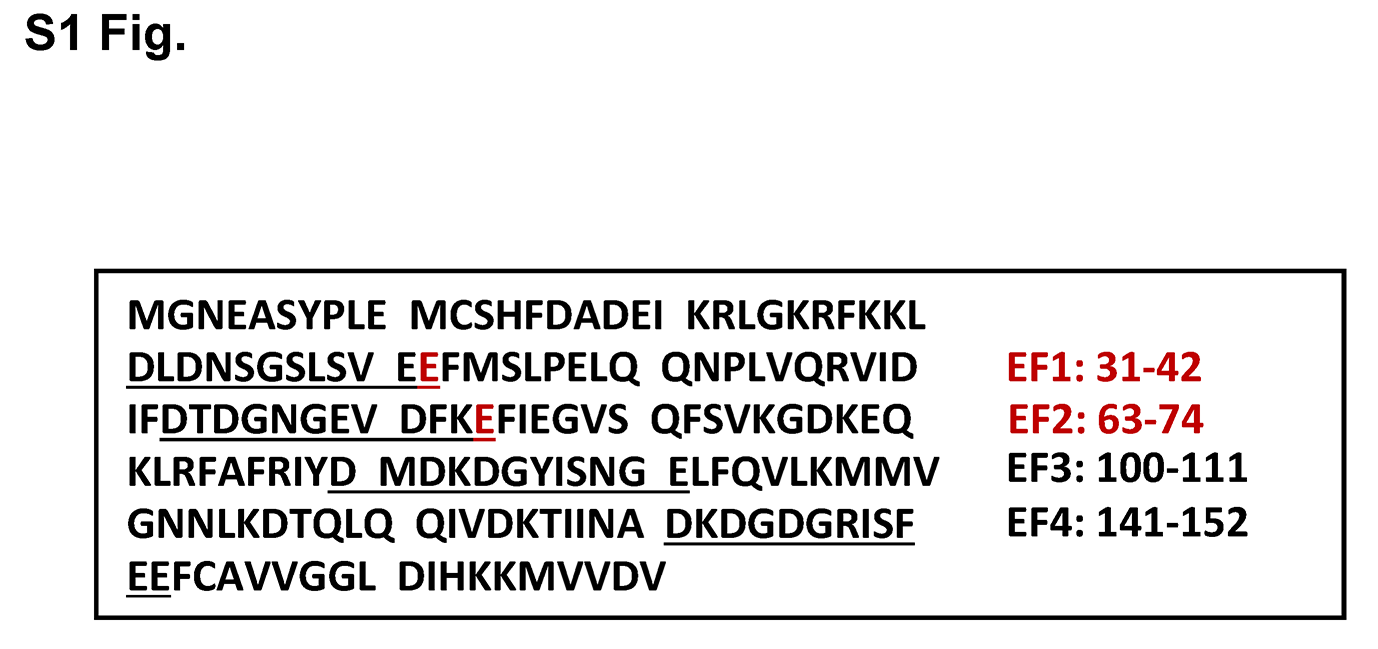

Supplement: S1 Fig — A Ca2+-binding-deficient CaN-B mutant was constructed using site-directed mutagenesis. Glutamic acid (E) in the 12th position of the 1st and 2nd EF-hand Ca2+-binding sites (shown in red characters) was replaced with lysine (K). (TIF) [file pone.0235360.s001.tif]

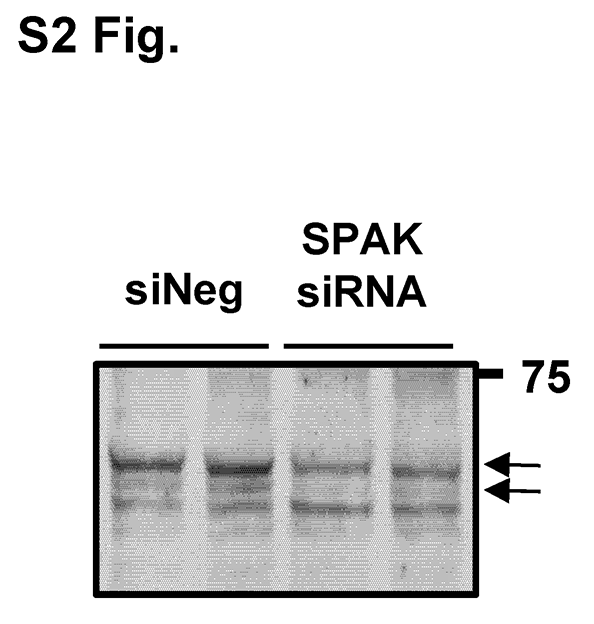

Supplement: S2 Fig — Immunoblots of phosphorylated SPAK in HEK293 cells with SPAK siRNA silencing and the negative control (siNeg). The disappearance of bands from cell lysates with SPAK siRNA silencing confirms the specificity of the antibody (shown with arrows). (TIF) [file pone.0235360.s002.tif]

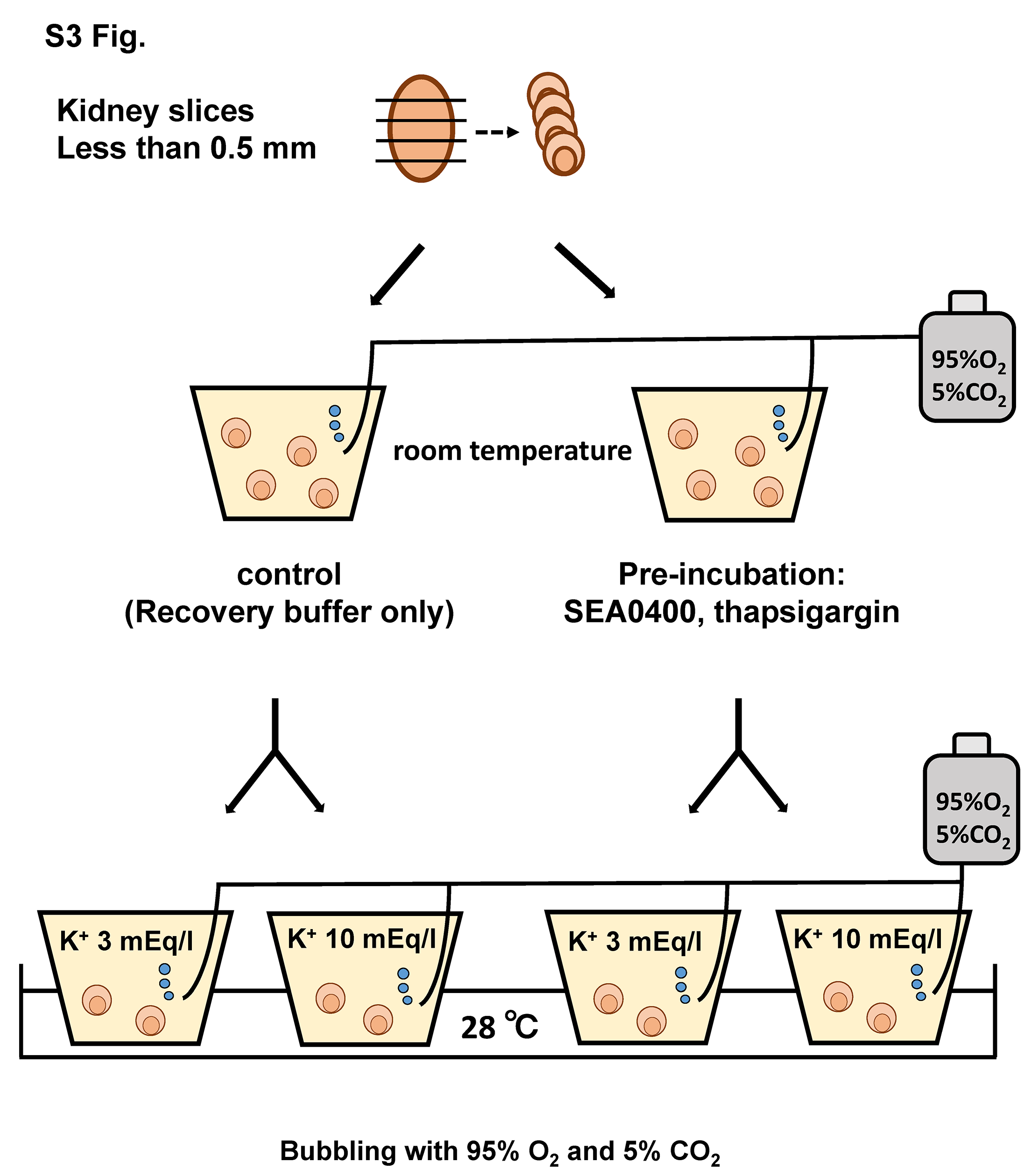

Supplement: S3 Fig — Kidneys were sliced into sections of <0.5 mm thickness. All solutions were continuously bubbled with 95% O2 and 5% CO2. Details are described in the Materials and Methods section. (TIF) [file pone.0235360.s003.tif]

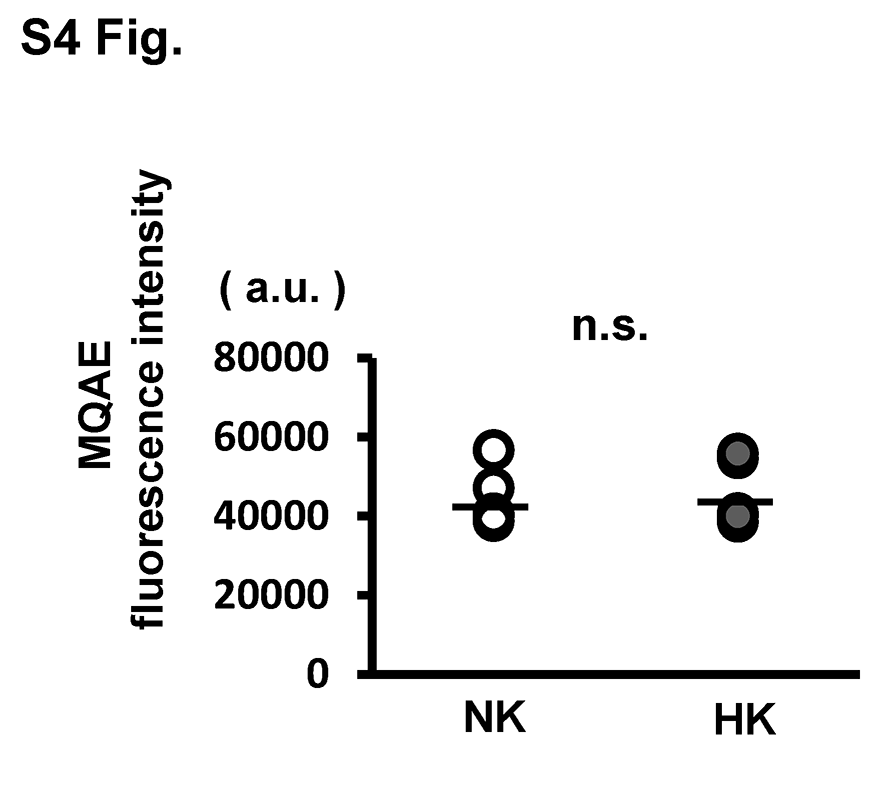

Supplement: S4 Fig — Intracellular [Cl−] was analyzed using the Cl−-sensing dye MQAE. High-K+ administration of 10 mM K+ did not show a significant change in intracellular [Cl−] compared with normal K+ administration of 3 mM K+. *represents significant differences at p <0.05 using an unpaired t-test. NK, normal potassium; HK, high potassium. (TIF) [file pone.0235360.s004.tif]

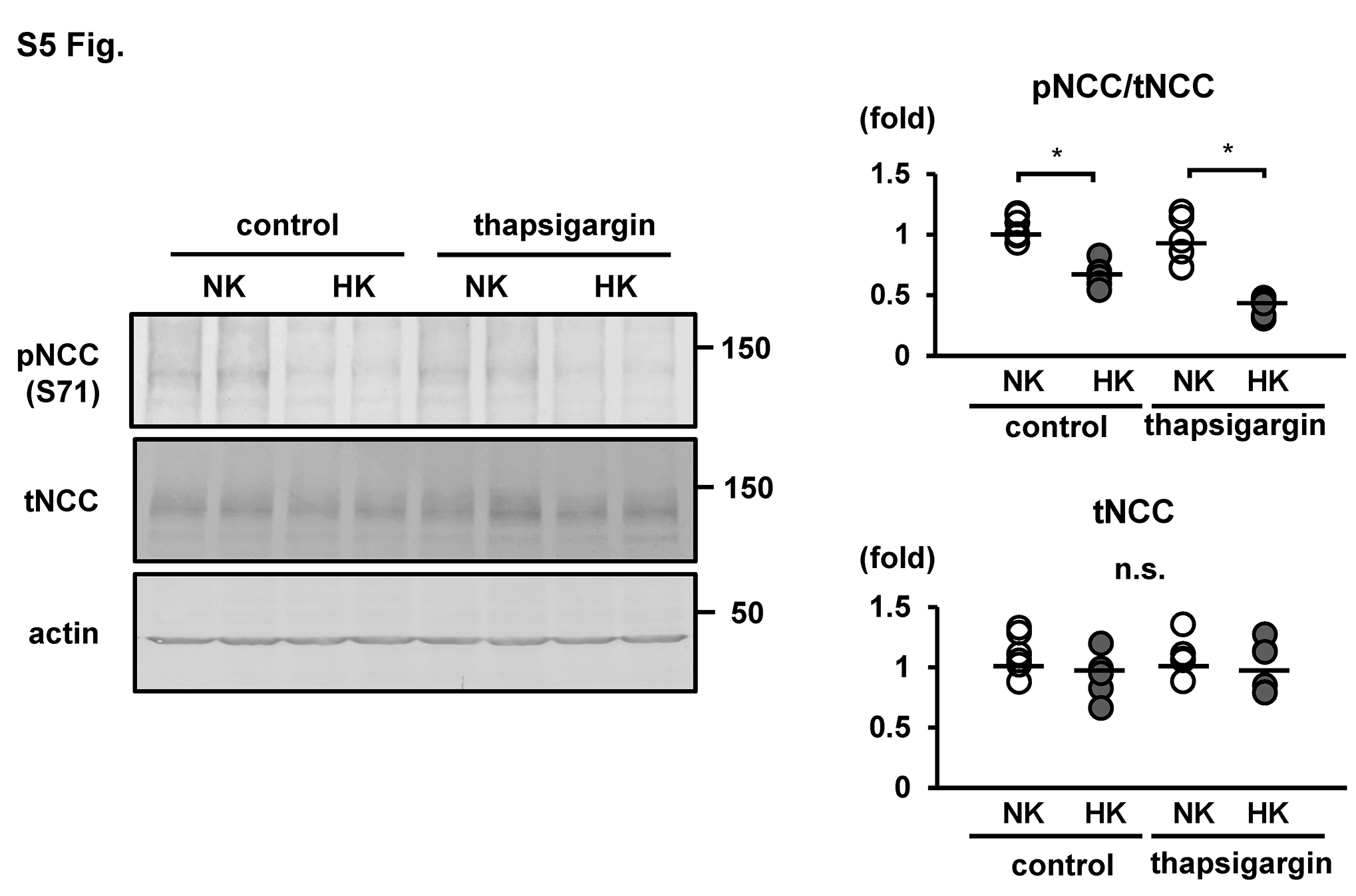

Supplement: S5 Fig — (left) Representative immunoblots of mouse kidney slices with 100 μM thapsigargin. The high-K+-induced reduction in the level of phosphorylated NCC was not inhibited in thapsigargin treatment. (right) Quantitative analysis of the total and phosphorylated NCC ratio in dot plots (n = 6). *p <0.05 by Tukey’s test after two-way ANOVA. NK, normal potassium; HK, high potassium. n.s., not significant. (TIF) [file pone.0235360.s005.tif]

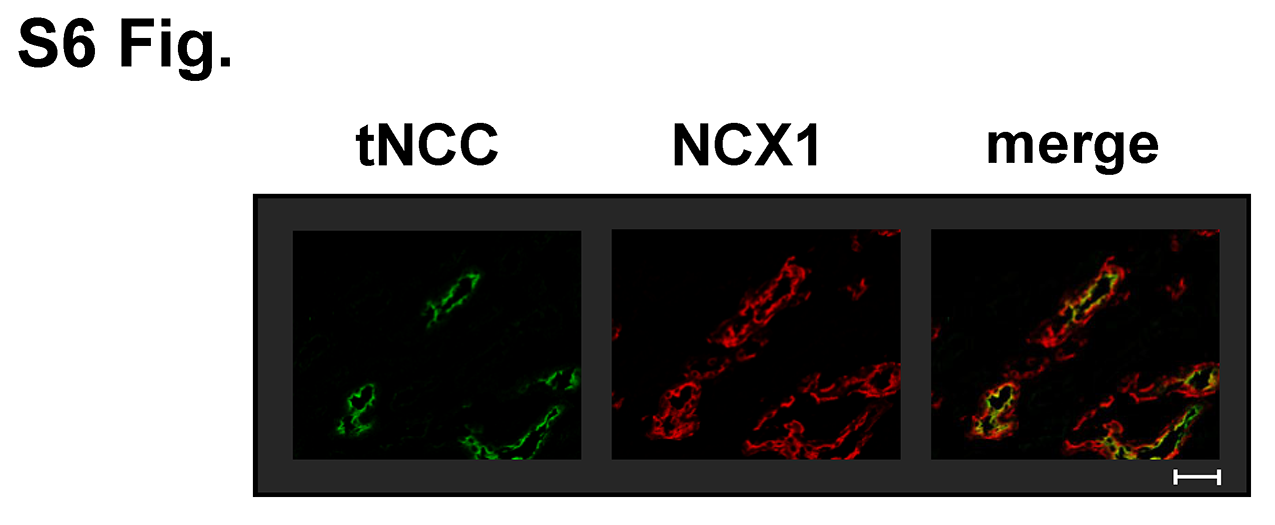

Supplement: S6 Fig — Double immunofluorescence of total NCC (green) and NCX1 (red) in mouse kidneys. NCC and NCX1 were co-localized in the distal convoluted tubule. Scale bars: 50 μm. (TIF) [file pone.0235360.s006.tif]

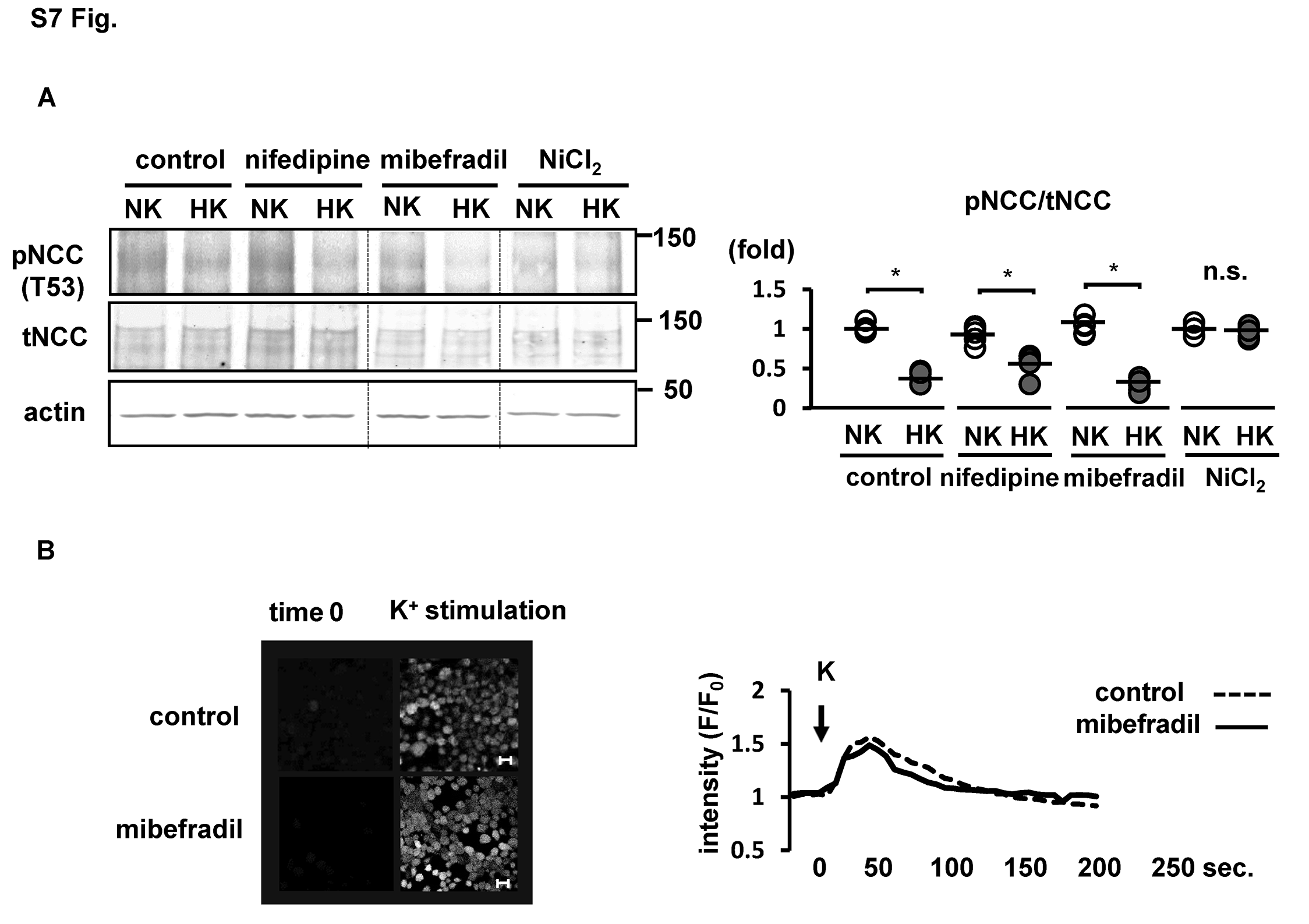

Supplement: S7 Fig — A. Evaluation of high-K+-induced NCC dephosphorylation in Flp-In NCC HEK293 cells treated with 1 μM nifedipine, 1 μM mibefradil, and 100 μM NiCl2. Normal K+ and high K+ are 3 mM and 10 mM, respectively. (right) Representative immunoblots. Although NiCl2 inhibited K+-induced NCC dephosphorylation, mibefradil and nifedipine did not. (right) Quantitative analysis of the total and phosphorylated NCC ratio in dot plots (n = 6). *represents significant differences at p <0.05 using Tukey’s test after a multiple-way ANOVA. NiCl2, nickel chloride; NK, normal potassium; HK, high potassium; n.s., not significant. B. The influx of Ca2+ after high- K+ stimulation in mibefradil treatment in Flp-In NCC HEK293 cells. (left) Representative images of Fluo 4 intensity 30 s after K+ administration. K+ (10 mM final concentration) was added to Flp-In NCC HEK293 cells 1 h after mibefradil treatment. The increased Fluo 4 fluorescence intensity following addition of K+ was not inhibited by mibefradil treatment. Scale bars, 20 μm. (right) Representative time-course of Fluo 4 fluorescence intensity. The x and y axes indicate time and Fluo 4 fluorescence intensity, respectively. (TIF) [file pone.0235360.s007.tif]

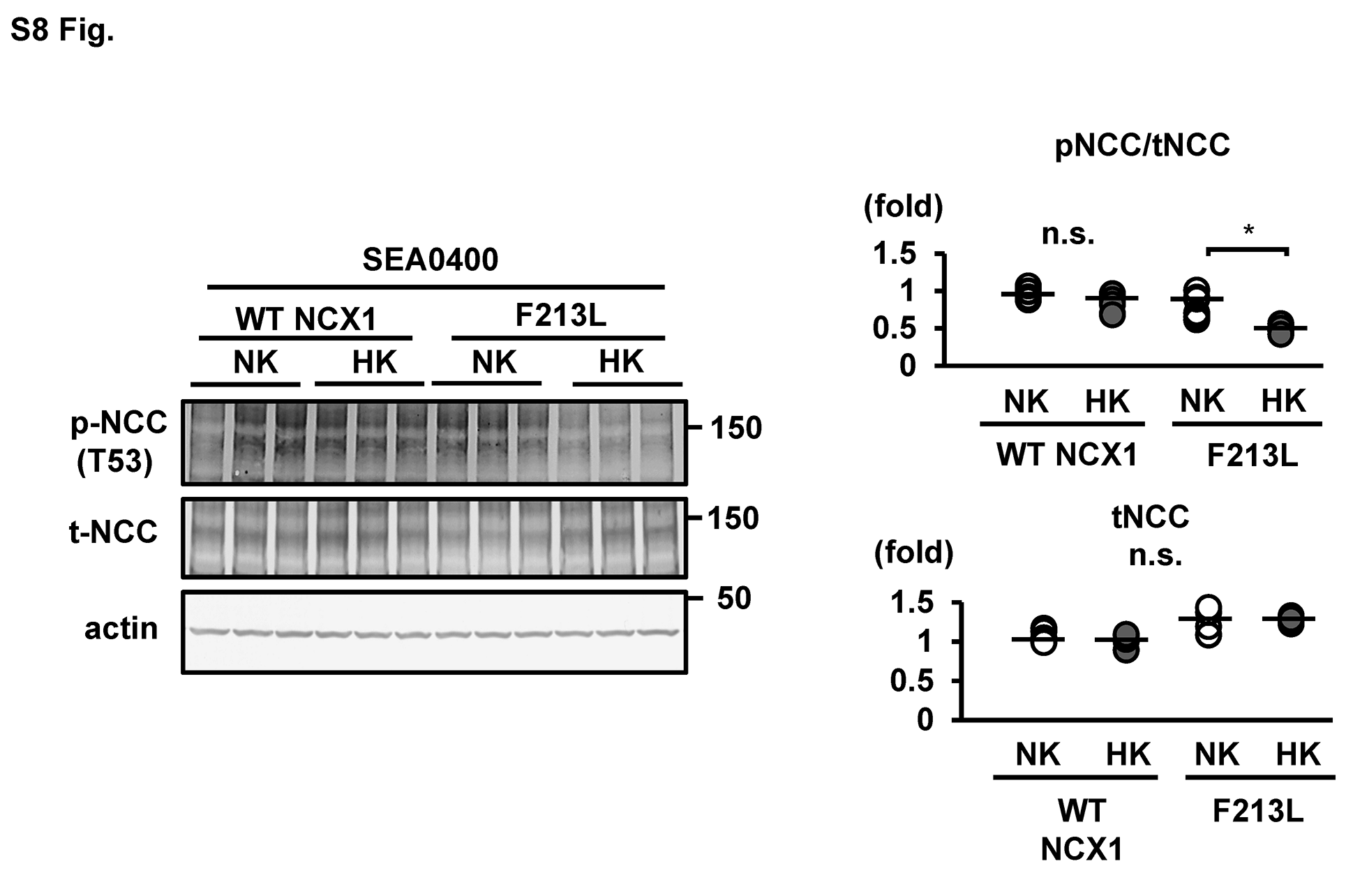

Supplement: S8 Fig — (left) Representative immunoblots of total and phosphorylated NCC in Flp-In NCC HEK293 cells overexpressing an NCX1 mutant (F213L) and wild-type NCX1. The inhibition of K+-induced NCC dephosphorylation with SEA0400 treatment was recovered in the cells overexpressing F213L NCX1. NK, normal potassium (K+ 3 mM); HK, high potassium (K+ 10 mM). (right) Quantitative analysis of the total and phosphorylated NCC ratio in column graphs (n = 6). *p < 0.05 by Tukey’s test after two-way ANOVA. (TIF) [file pone.0235360.s008.tif]

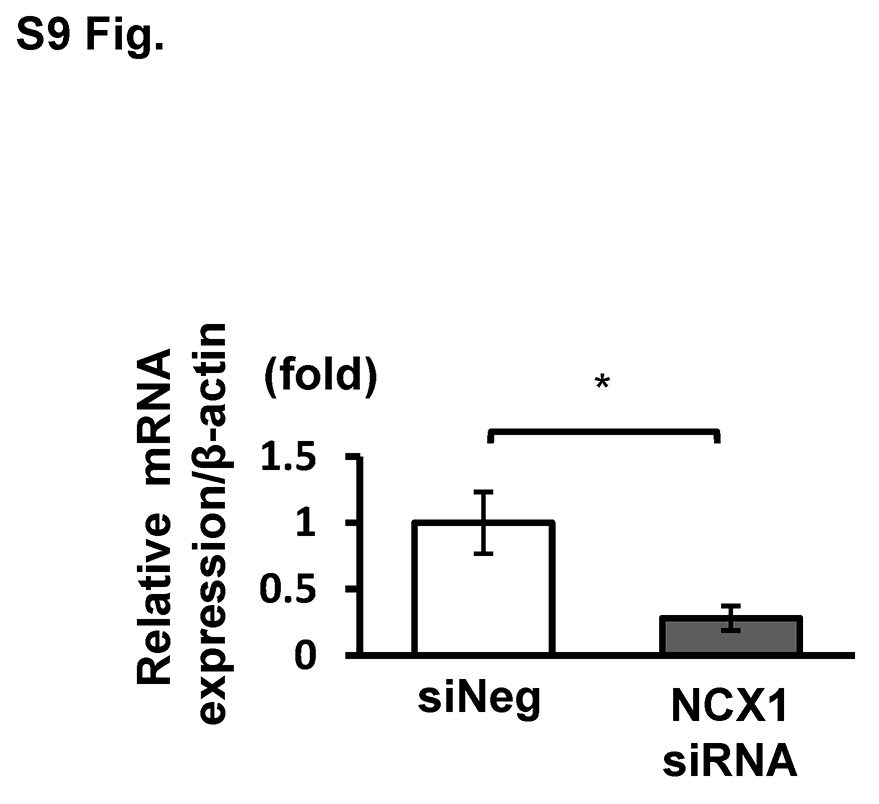

Supplement: S9 Fig — Relative mRNA expression of NCX1 in Flp-In NCC HEK293 cells following NCX1 siRNA silencing was significantly decreased compared with that in cells with negative control siRNA. n = 6. Means ± standard errors of the mean. *represents significant differences at p <0.05 using an unpaired t-test. (TIF) [file pone.0235360.s009.tif]

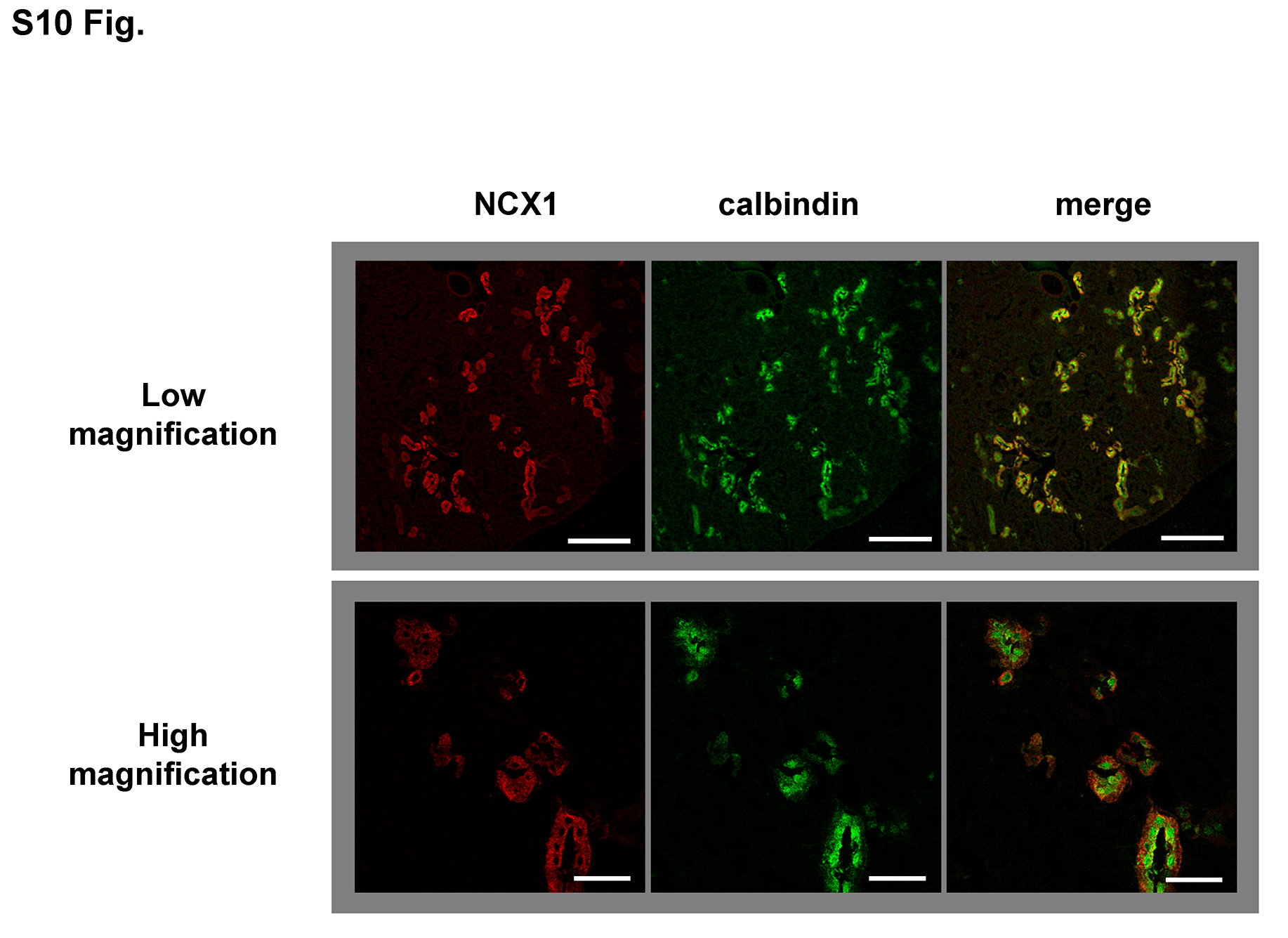

Supplement: S10 Fig — Confirmation of the localization of NCX1 and calbindin in wild-type mouse kidney. Red: NCX1, Green: calbindin. Scale bars indicate 50 μm and 200 μm in high and low magnification images, respectively. (TIF) [file pone.0235360.s010.tif]

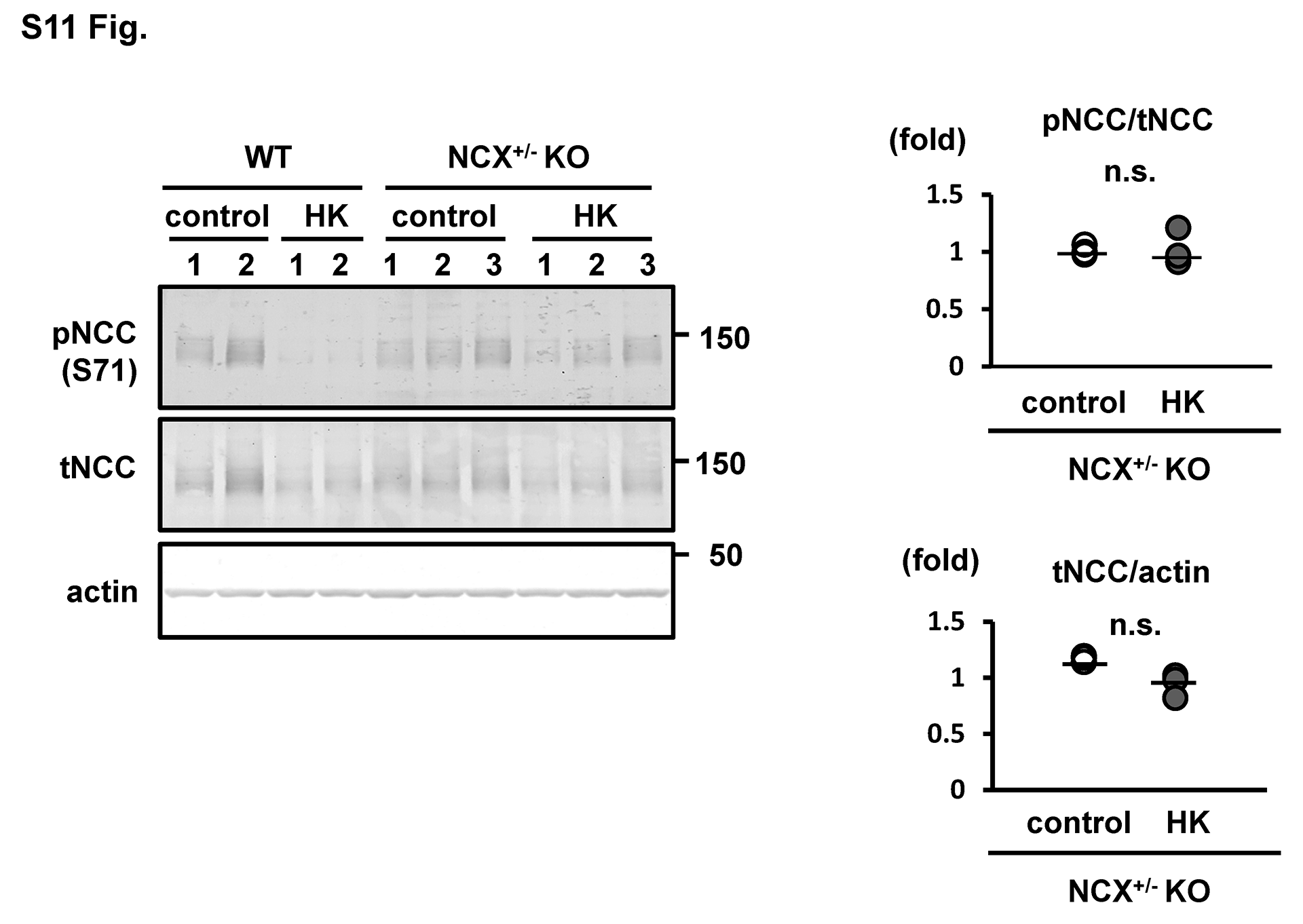

Supplement: S11 Fig — (left) Immunoblots of total NCC and phosphorylated NCC in NCX+/− KO mice and wild-type mice. A rapid decrease in the level of phosphorylated NCC after K+ administration was not evident in NCX+/− KO mice. (right) Quantitative analysis of total and phosphorylated NCC in NCX+/− KO mice shown in column graphs (n = 3). * represents significant differences at p <0.05 using an unpaired t-test. (TIF) [file pone.0235360.s011.tif]

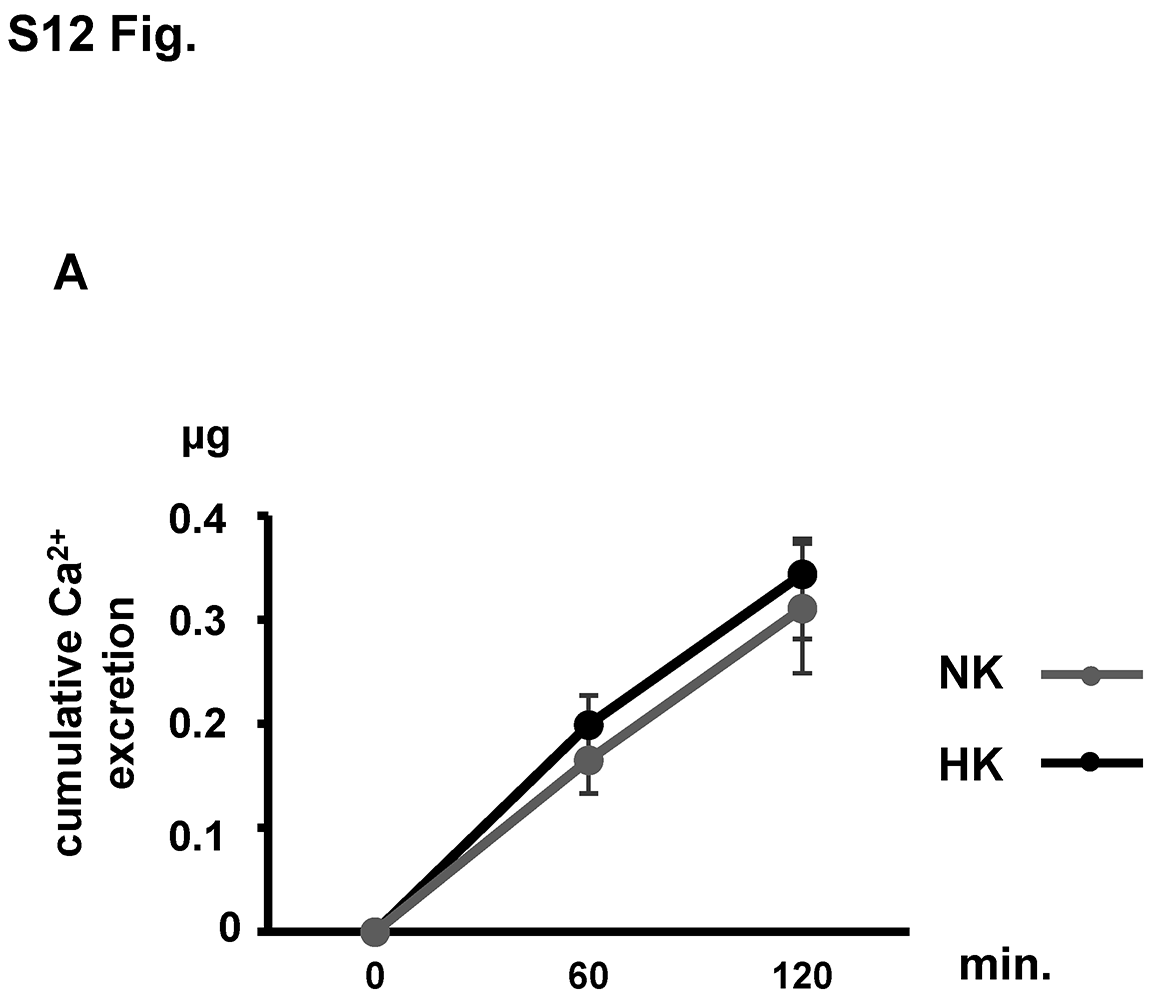

Supplement: S12 Fig — Urinary Ca2+ excretion was not different between mice administered high K+ and those administered normal K+ (n = 6). Means ± SEM. *p <0.05 by unpaired t-test at each time point. (TIF) [file pone.0235360.s012.tif]

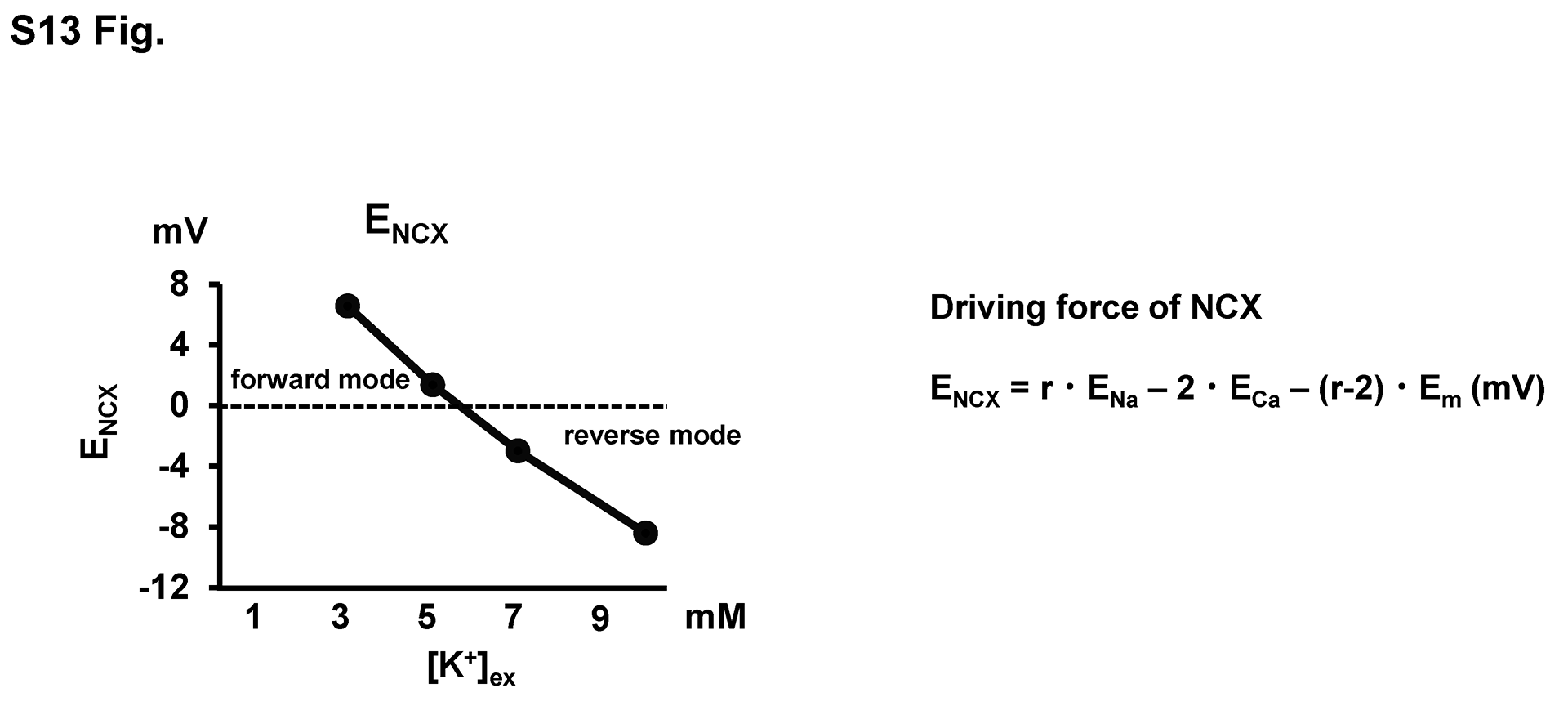

Supplement: S13 Fig — NCX drives in forward-mode and reverse-mode under low- and high-K+ conditions, respectively. ENCx, driving force of NCX. (TIF) [file pone.0235360.s013.tif]

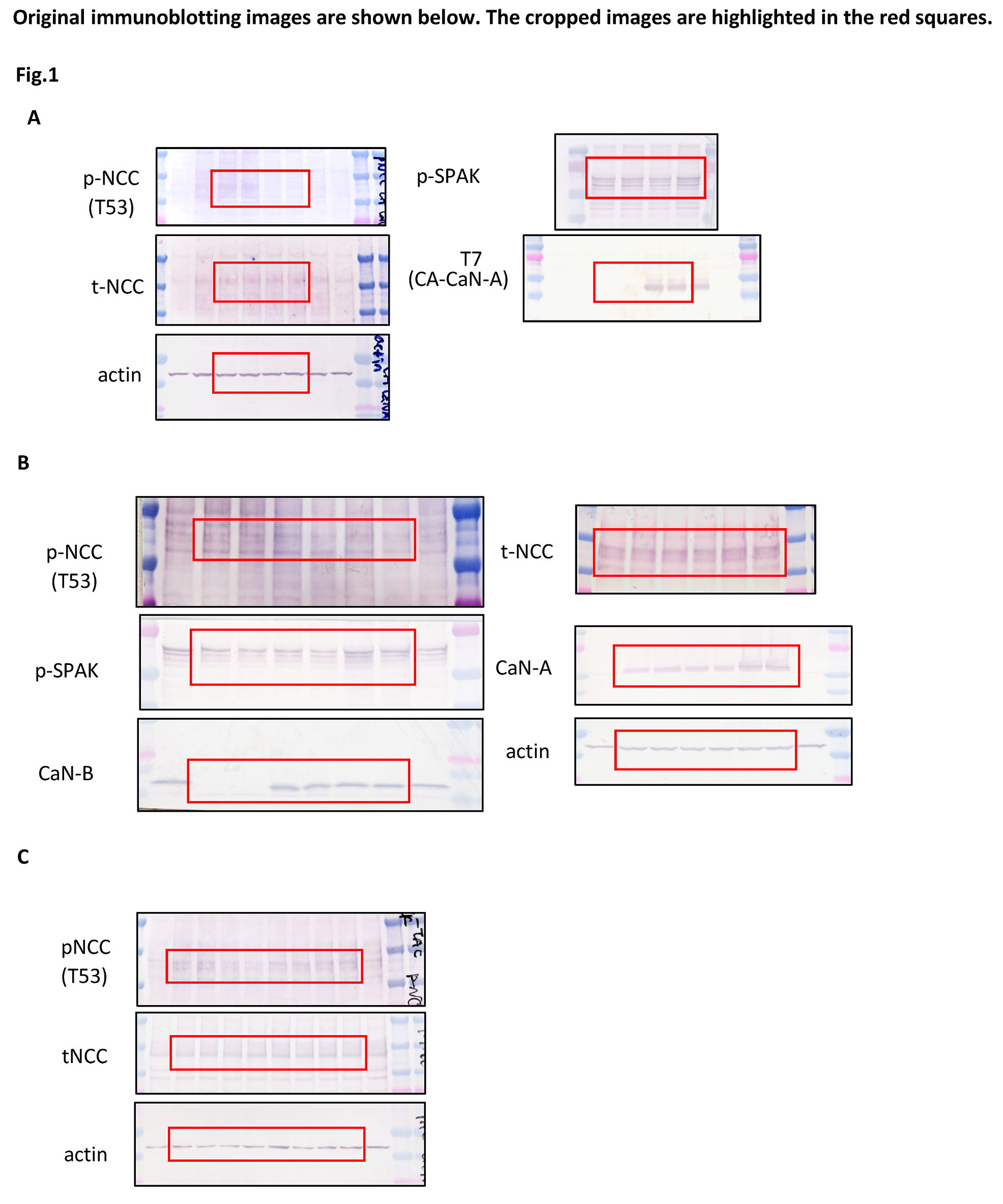

Supplement: S1 Raw image — (TIF) [file pone.0235360.s014.tif]

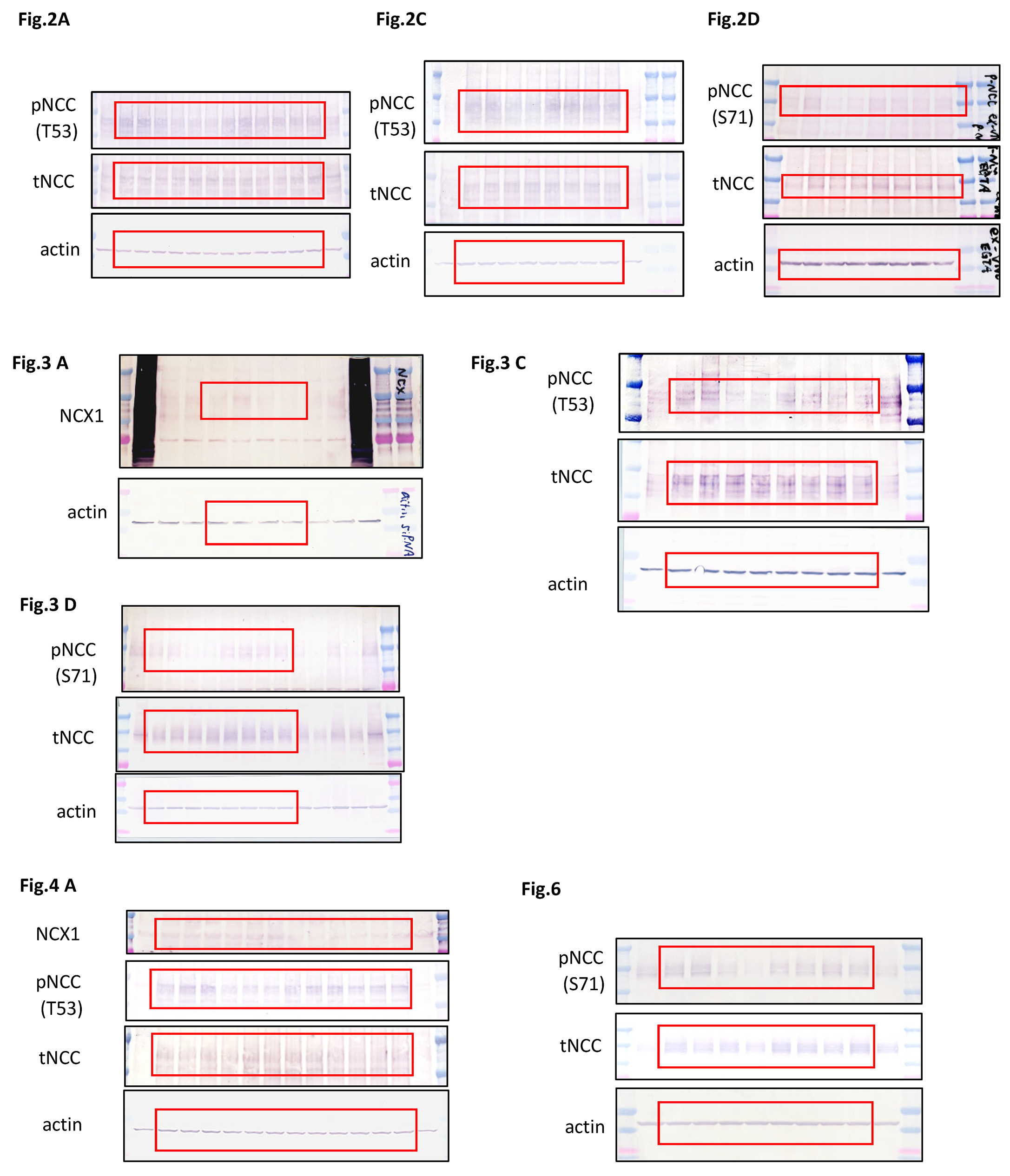

Supplement: S2 Raw image — (TIF) [file pone.0235360.s015.tif]

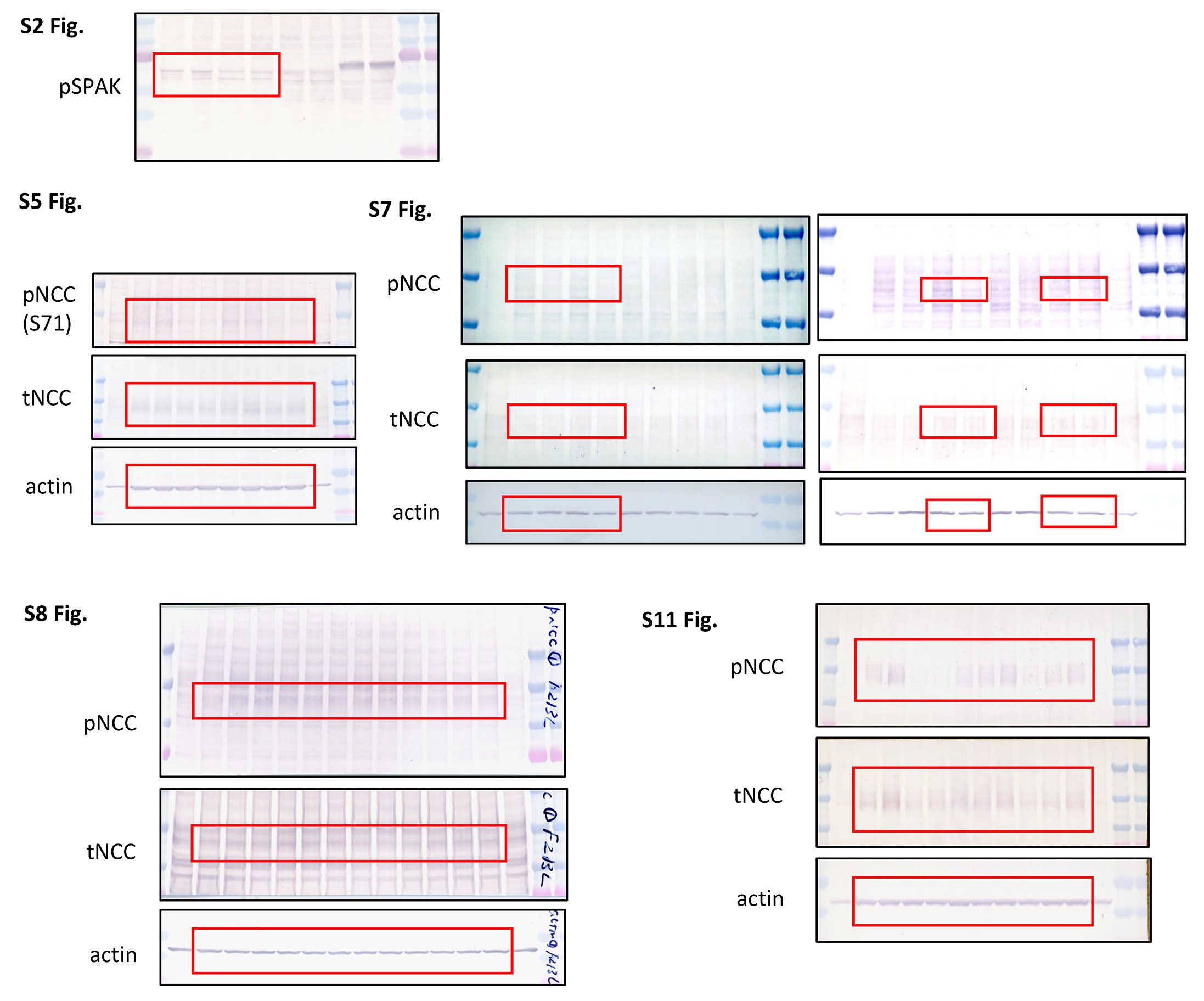

Supplement: S3 Raw image — (TIF) [file pone.0235360.s016.tif]
