# Supplementary material for: Sodium–calcium exchanger 1 is the key molecule for urinary potassium excretion against acute hyperkalemia
Source: PLoS One. 2020 Jun 30;15(6):e0235360. doi: 10.1371/journal.pone.0235360 (PMC7326190; doi:10.1371/journal.pone.0235360)
Supplement: S1 File — (DOCX) [file pone.0235360.s017.docx]

**S1 Materials and methods**

Details of Western blotting and immunofluorescence, the NCX1 heterozygous knockout mice (NCX^+/−^ KO) experiments, and quantitative RT–PCR are described in the S1 Materials and Methods.

**Western blotting and immunofluorescence**

For the Western blotting, the kidney samples were lysed in a detergent-free buffer of 250 mM sucrose, 10 mM triethanolamine, 1 mM EGTA, 1 mM EDTA, 1 mM Na orthovanadate, 50 mM Na fluoride, and complete protease inhibitor cocktail (Roche) with a homogenizer. Then, the homogenates were centrifuged to separate the extranuclear fraction into a whole kidney lysate (600 *g* supernatant) and a crude membrane fraction (17,000 *g* pellet). The samples were mixed with ß-ME Sample Treatment for Tris SDS (Cosmo Bio, Tokyo, Japan) and incubated for 20 min at 60°C. Protein concentration was determined using a Bradford ULTRA kit (Expedeon, UK). The protein samples were separated using SDS-PAGE on 5%–20% gradient gels in Tris-Glycine-SDS running buffer (Takara Bio Inc., Japan). Then the proteins were transferred to nitrocellulose membranes in a transfer buffer of 100 mM Tris and 192 mM Glycine. Next, the membranes were blocked with 5% skim milk in TBST for 30 min at room temperature. Then, the membranes were incubated with a primary antibody (see Table 1) overnight at 4°C. After three washes with TBST, the membranes were incubated with a secondary antibody (see Table 1) for 45 min at room temperature. After three washes with TBST, Western Blue (Promega) was used to detect the signals of the proteins. The relative intensities of the detected protein bands were analyzed and quantified using ImageJ software (National Institutes of Health).

For the immunofluorescence analysis, the kidney samples were fixed by perfusion through the left ventricle using periodate lysine (0.2 M) and paraformaldehyde (2%) in PBS. Then, the tissue samples were soaked for several hours in 20% sucrose in PBS, embedded in Tissue Tek O.C.T. Compound (Sakura Finetechnical Co., Ltd, Tokyo, Japan) and frozen in liquid nitrogen. The antibodies used for immunofluorescence are described in Table 1.

**Quantitative real-time reverse transcription–polymerase chain reaction**

Total RNA was extracted from Flp-In NCC HEK293 cells with siRNA and negative control siRNA using Sepasol-RNA I Super G (Nacalai Tesque, Kyoto, Japan) green monophasic solution, according to the manufacturer’s protocol. cDNA was synthesized using a ReverTra Ace^®^ (Toyobo Co., Ltd., Osaka, Japan) qPCR RT kit. Quantitative real-time RT–PCR was performed using TB Green Premix Ex Taq II (Cat # RR820A, Takara Bio Inc., Shiga, Japan) reagent and Thermal Cycler Dice Real Time System Lite (Takara Bio Inc., Shiga, Japan) software. All reactions were performed in duplicate, and the relative mRNA expression level of each target gene was normalized to that of β-actin as an internal control. The primers used for RT–PCR are shown in Supplemental Table 1.

**S1 Table. Primer sets used for study**

|  | Primer sequences (5’- 3’) for RT–PCR and Quantitative RT–PCR |
| --- | --- |
| NCX1 | CATTGAAGCTATCACTGTCAG (fwd) |
| (human) | CCATTCCAGTATTCAG TAGG (rev) |
| β-actin | TGGCATTGCCGACAGGATGC (fwd) |
| (human) | TCCACACGGAGTACTTGCGC (rev) |
|  | Primer sequences (5’- 3’) for mutagenesis |
| CaN-B (EF1) | GTGGAAAAGTTCATGTCTCTGCCTGAG (fwd) |
| (human) | CATGAACTTTTCCACACTCAAAGAACC (rev) |
| CaN-B (EF2) | TTTAAAAAATTCATTGAGGGCGTCTCT (fwd) |
| (human) | AATGAATTTTTTAAAGTCTACTTCTCC (rev) |
| NCX1 (F213L) | AGCATCTTAGCCTACACCTGGCTTTAC (fwd) |
| (human) | GTAGGCTAAGATGCTCCAGGCTGCTGTC (rev) |
|  | Primer sequences (5’- 3’) for subcloning |
| CaN-A | TGGACAGCAAATGGGTCGCGAATTCATGTCCGAGCCCAAGGC (fwd) |
| (human) | GGCGGCCAAGCTTCTGCAGGTCGACTCACTGAATATTGCTGC (rev) |
| CaN-B | TGGACAGCAAATGGGTCGCGAATTCATGGGAAATGAGGCAAGTTATC (fwd) |
| (human) | GGCGGCCAAGCTTCTGCAGGTCGACTCACACATCTACCACCATC (rev) |
| CA-CaN-A | TGGACAGCAAATGGGTCGCGAATTGATGTCCGAGCCCAAGGCGAT (fwd) |
| (mouse) | GGCGGCCAAGCTTCTGCAGGTCGACTCAGGCTGCGGCCGTGGCTC (rev) |
| NCX1 (WT) | AAGCTTCTGCAGGTCGACTTAGAAGCCTTTTATGTGGC (fwd) |
| (human) | CAAATGGGTCGCGAATTCATGTACAACATGCGGCGAT (rev) |

**Sodium-calcium exchanger 1 heterozygous knockout mouse (NCX^+/−^ KO) experiments**

Experiments were performed on male adult NCX^+/−^ KO mice weighing 25–30 g. All mice were housed under diurnal lighting conditions with a light period from 8:00 a.m. to 8:00 p.m. Food was removed 2 h prior to K^+^ oral gavage. A 2 ml saline solution was intraperitoneally injected 1 h prior to K^+^ load, instead of allowing access to water. A volume of 15 µl/g of body weight of high-K^+^ solution, of 1.7% K^+^ in 2% sucrose, or a control solution, of 2% sucrose, was administered to mice using oral gavage. The kidneys were collected 15 min after oral gavage.
