# Supplementary material for: Sodium–calcium exchanger 1 is the key molecule for urinary potassium excretion against acute hyperkalemia
Source: PLoS One. 2020 Jun 30;15(6):e0235360. doi: 10.1371/journal.pone.0235360 (PMC7326190; doi:10.1371/journal.pone.0235360)
Supplement: Attachment — Submitted filename: For the editor - PONE-D-19-36071.pdf [file pone.0235360.s018.pdf]

UMR_S 1155
Unité mixte Inserm-Université
MALADIES RENALES FREQUENTES ET RARES:
DES MECANISMES MOLECULAIRES A LA MEDECINE PERSONNALISEE

Directeur : Dr CHRISTOS CHATZIANTONIOU

Paris, the 8th of April 2020,

Object: Review for PONE-D-19-36071

Dear Prof. Dussaule,

The manuscript PONE-D-19-36071, entitled 'Sodium–calcium exchanger 1 is the key molecule for urinary potassium excretion against acute hyperkalemia', by Shoda and co-authors, describes the characterisation of the crucial role played by the Na-Ca exchanger NCX1 in the regulation of the activity of the Na-Cl cotransporter NCC in the distal nephron during a K-load.

The paper is well written. The data are solid and support the hypotheses and conclusions drawn. I have a few comments, which you will find below. Therefore, I recommend that the paper should be revised, with major revisions.

Best regards,
Juliette Hadchouel, VetMD, PhD

Review for PONE-D-19-36071

The manuscript PONE-D-19-36071, entitled 'Sodium–calcium exchanger 1 is the key molecule for urinary potassium excretion against acute hyperkalemia', by Shoda and co-authors, describes the characterisation of the crucial role played by the Na-Ca exchanger NCX1 in the regulation of the activity of the Na-Cl cotransporter NCC in the distal nephron during a K-load.

Overall, the results described here support the hypotheses and the conclusions drawn. However, I have the following comments.

Major comments:

Material and Methods – Animal experiment : why were the kidneys collected 15 minutes after the oral K gavage for protein extraction? The metabolic data were collected every 30 minutes after the oral gavage. Why is it different?

Results :

(1) Figure 1 : Calcineurin is essential for K+-induced NCC dephosphorylation

- I don't understand the result and relevance of the experiment describing the consequence of the overexpression of CaN-A on NCC phosphorylation. How could CaN-A work without CaN-B, which is barely expressed in HEK293 cells, as explained by the authors in the next paragraph? Conversely, why did the authors overexpress CaN-A when transfecting CaN-B is sufficient to induce NCC dephosphorylation (Fig 1B)?

(2) Figure 2D: why did the authors quantify pNCC-S71 in kidney slices and mice rather than NCC-p53, used *in vitro*?

In addition, the figures would be easier to read if the system in which the experiments are conducted were indicated on the figure.

(3) Figure S11: why did the authors quantify the pNCC/tNCC ratio rather than pNCC and tNCC separately like in the other experiments?

(4) Table 2: 'Blood K+ level at 120 min following a high-K+ load was significantly higher in SEA0400-treated mice than in vehicle-treated counterparts (Table 2)'.

In their previous study, using tacrolimus to inhibit calcineurin, the authors did not observe a change in plasma potassium in tacrolimus-treated animals even though NCC dephosphorylation was inhibited. Could the authors comment on the difference in plasma K between tacrolimus- and SEA0400-treated animals?

(5) Have the authors studied the role of NCX1 after a longer exposure to high K ?

Minor comments:

- Abstract : the authors should remove the following sentence, which is not at the correct place : ‘The mice were housed in metabolic cages for urine sample collection’.

- Introduction, page 6 : the reference’s number in the following sentence is incorrect: ‘In our previous study, we observed that the CaN inhibitor, tacrolimus, …urinary K+ excretion in the acute phase[22]’. The authors should check all the other references’ number.

- Results: Figures 3 and 5 should be merged.
