# Supplementary material for: Sodium–calcium exchanger 1 is the key molecule for urinary potassium excretion against acute hyperkalemia
Source: PLoS One. 2020 Jun 30;15(6):e0235360. doi: 10.1371/journal.pone.0235360 (PMC7326190; doi:10.1371/journal.pone.0235360)
Supplement: Attachment — Submitted filename: Comments for authors PONE-D-19-36071.pdf [file pone.0235360.s019.pdf]

Review for PONE-D-19-36071

The manuscript PONE-D-19-36071, entitled 'Sodium–calcium exchanger 1 is the key molecule for urinary potassium excretion against acute hyperkalemia', by Shoda and co-authors, describes the characterisation of the crucial role played by the Na-Ca exchanger NCX1 in the regulation of the activity of the Na-Cl cotransporter NCC in the distal nephron during a K-load.

Overall, the results described here support the hypotheses and the conclusions drawn. However, I have the following comments.

Major comments:

Material and Methods – Animal experiment : why were the kidneys collected 15 minutes after the oral K gavage for protein extraction? The metabolic data were collected every 30 minutes after the oral gavage. Why is it different?

Results :

(1) Figure 1 : Calcineurin is essential for K+-induced NCC dephosphorylation

- I don't understand the result and relevance of the experiment describing the consequence of the overexpression of CaN-A on NCC phosphorylation. How could CaN-A work without CaN-B, which is barely expressed in HEK293 cells, as explained by the authors in the next paragraph? Conversely, why did the authors overexpress CaN-A when transfecting CaN-B is sufficient to induce NCC dephosphorylation (Fig 1B)?

(2) Figure 2D: why did the authors quantify pNCC-S71 in kidney slices and mice rather than NCC-p53, used *in vitro*?

In addition, the figures would be easier to read if the system in which the experiments are conducted were indicated on the figure.

(3) Figure S11: why did the authors quantify the pNCC/tNCC ratio rather than pNCC and tNCC separately like in the other experiments?

(4) Table 2: 'Blood K+ level at 120 min following a high-K+ load was significantly higher in SEA0400-treated mice than in vehicle-treated counterparts (Table 2)'.

In their previous study, using tacrolimus to inhibit calcineurin, the authors did not observe a change in plasma potassium in tacrolimus-treated animals even though NCC dephosphorylation was inhibited. Could the authors comment on the difference in plasma K between tacrolimus- and SEA0400-treated animals?
